# Initiating Ullmann-like coupling of Br_2_Py by a semimetal surface

**DOI:** 10.1038/s41598-021-82973-z

**Published:** 2021-02-09

**Authors:** Jinping Hu, Jinbang Hu, Hongbing Wang, Kongchao Shen, Huan Zhang, Chaoqin Huang, Lei Xie, Qiwei Tian, Han Huang, Zheng Jiang, Fei Song

**Affiliations:** 1 Key Laboratory of Interfacial Physics and Technology, Shanghai Institute of Applied Physics, Chinese Academy of Sciences, Shanghai, 201000 China; 2 University of Chinese Academy of Sciences, Beijing, 100100 China; 3 Department of Physics, Center of Quantum Spintronics, Norwegian University of Science and Technology, 7491 Trondheim, Norway; 4 Shanghai Synchrotron Radiation Facility, Zhangjiang Laboratory, Shanghai Advanced Research Institute, Chinese Academy of Sciences, Shanghai, 201000 China; 5 School of Physics Science and Electronics, Central South University, Changsha, 410083 China; 6 Present Address: Institute of Functional Nano & Soft Materials (FUNSOM), Soochow University, Suzhou, 215123 China

**Keywords:** Scanning probe microscopy, Surface assembly, Surfaces, interfaces and thin films

## Abstract

Intensive efforts have been devoted to surface Ullmann-like coupling in recent years, due to its appealing success towards on-surface synthesis of tailor-made nanostructures. While attentions were mostly drawn on metallic substrates, however, Ullmann dehalogenation and coupling reaction on semimetal surfaces has been seldom addressed. Herein, we demonstrate the self-assembly of 2, 7-dibromopyrene (Br_2_Py) and the well controllable dehalogenation reaction of Br_2_Py on the Bi(111)–Ag substrate with a combination of scanning tunnelling microscopy (STM) and density functional theory calculations (DFT). By elaborately investigating the reaction path and formed organic nanostructures, it is revealed that the pristinely inert bismuth layer supported on the silver substrate can initiate Ullmann-like coupling in a desired manner by getting alloyed with Ag atoms underneath, while side products have not been discovered. By clarifying the pristine nature of Bi–Ag(111) and Ullmann-like reaction mechanisms, our report proposes an ideal template for thoroughly exploring dehalogenative coupling reaction mechanisms with atomic insights and on-surface synthesis of carbon-based architectures.

## Introduction

Bottom-up building of covalently bound organic architectures with well-defined arrangements is key to surface physics and surface chemistry towards molecular electronics and nanodevices^1–5^. By predesigning chemical structures of precursors, the on-surface synthesis strategy harvests appealing successes at fabricating tailor-made nanostructures, for example, artificial graphene nanoribbons, graphene ring, polyacenes and so on^6–12^. Particularly, Ullmann-like coupling or namely, the dehalogenative reaction of reactants on surface has been recognized as an extremely efficient and robust approach towards constructing stable covalent molecular architectures on demand^13–21^. Despite of these encouraging progresses, on-surface Ullmann-like coupling has been achieved mostly on metallic substrates which act as the heterogeneous catalyst for the coupling reactions, while investigations on relatively inert surfaces or insulators are minor. On the other hand, electrically insulating substrates are crucial to sufficiently decouple the molecular structures from substrate towards future applications in nanoelectronics. Consequently, the rational synthesis of covalent nanostructures directly on less reactive or inert surfaces is essentially meaningful.

**Figure 1:**
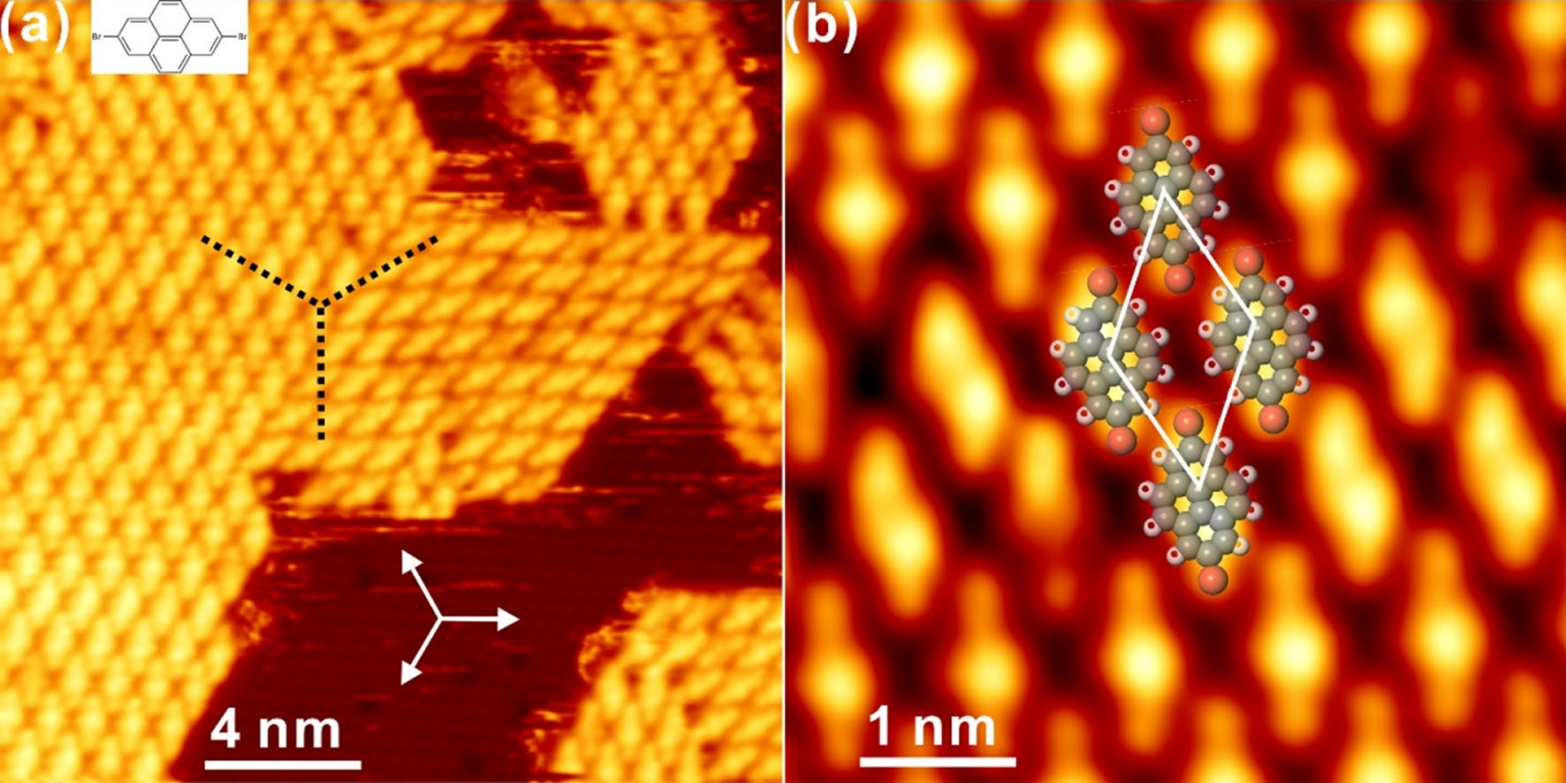
**(a)** A typical large-scale STM topology recorded after the deposition of Br_2_Py onto the Bi–Ag(111) substrate at RT. **(b)** A close-up STM view of **(a)** with the unit cell of self-assembly superimposed. Scanning parameters for **(a)** and **(b)**: U_bias_ = − 1.8 V, I_t_ = 0.2 nA. Colour code: C, grey; H, white; Br, brown.

In this context, attention has thus been paid to the surface Ullmann-like coupling supported on non-metallic substrates, for instance, on top of the graphene and boron nitride thin layer decoupled from the nickel substrate^22–24^, on a bulk insulator^25,26^ and metal oxide surfaces^27–30^. However, due to the shortage of catalytic activity during the dehydrogenation reaction, these attempts seem to be not so satisfactory as expected with the sacrifice of reaction selectivity after the high-temperature annealing, for instance. Thus, on-surface Ullmann-like coupling reaction based on non-metallic surfaces requires further dedicated explorations for the fabrication of tailored nanoarchitectures, and the substrate catalytic activity also needs to be properly considered. Bismuth, known as a typical semimetal with the reduced surface activity, was introduced recently in the form of Bi-metal complex into both the molecular self-assembly and on-surface reaction^31–33^, whereas the catalytic performance of bismuth was still under debate, to some extent. For instance, it was demonstrated that the DBBA precursor got completely desorbed on the bismuth decorated Au(111) surface and therefore no Ullmann-like coupling was observed^31^. On the other hand, the Bi(111)–Au substrate was revealed to successfully initiate the metalation of phthalocyanine^33^. As demonstrated in literature, the growth mode of Bi on Au(111) differs significantly from that on Ag(111). Au(111) solely supports the atomic-layer growth of Bi atop with the honeycomb structure, while bismuth prefers to form surface alloy by incorporating with Ag atoms after adsorption on Ag(111)^34,35^, which introduces rich electronic states around the Fermi level^36^ and might thus effectively promote dehalogenation and homocoupling reactions. In this regard, a bismuth covered silver surface, namely, Bi/Ag(111), is chosen in this report to investigate whether the surface Ullmann-like coupling can be initiated by thermal annealing. A symmetrically halogenated precursor, 2,7-dibromopyrene, Br_2_Py^37^ (C_16_H_8_Br_2_, as shown in Fig. 1) is chosen as the precursor. By revealing the structural evolution of organic nanostructures at the atomic level via Scanning tunnelling microscopy (STM), we demonstrate herein that the thin bismuth layer atop silver presents the promising catalytic activity towards on-surface dehalogenation, and the stepwise Ullmann-like coupling reaction is clearly revealed and comprehensively elucidated thanks to the controllable dehalogenation reaction on the Bi/Ag(111) substrate.

## Results and discussion

Thin bismuth layer with the (111) facet was prepared on top of a clean Ag(111) substrate prior the deposition of Br_2_Py, and detailed information regarding on the preparation procedure can be found in the experimental section and Fig. S1 in the Electronic Supplementary Material (ESM). Initial deposition of Br_2_Py onto the Bi–Ag(111) surface held at room temperature (RT) results in the formation of well-ordered domains on terraces of the Bi–Ag(111) surface, as indicated by the STM topology in Fig. 1a. Apparently, the Br_2_Py precursors are kept intact after adsorption on Bi–Ag(111), while molecules assembled into closed packed structures that display three azimuthal domains on surface in relation to the substrate’s three-fold symmetry (as indicated by black dashed lines in Fig. 1(a)). Further inspection reveals that the molecular axis is in principle aligned at 30° rotation with respect to the high symmetry directions from the Bi(111) substrate (marked with white arrows), suggesting symmetry inheriting from substrate to the assembled Br_2_Py array and the delicate mediating between the molecule–substrate and intermolecular interactions. The zoom-in STM image in Fig. 1b indicates that Br_2_Py precursors are arranged parallel forming a rhombic unit cell with lattice parameters of a = b = 11.0 ± 0.2 Å and α = 60 ± 1°, which is in general consistent with previous reports of Br_2_Py adsorbed on the metal substrate^41,42^, whereas the self-assembly arrangement is stabilized by both the halogen bonding (–C–Br…Br–C–) and hydrogen bonding (–C–Br…H–C–) motifs between neighbouring molecules. Herein, the weak molecule–substrate interaction is responsible for the rotational alignment of the molecules with respect to the principal directions of substrate^38–40^, while the intermolecular halogen bonding and hydrogen bonding motifs between Br_2_Py monomers are responsible for the formation of the stable self-assembly phase^41,42^. Nevertheless, dark holes are also visible on surface in Fig. 1a, which are assigned to defects embedded in the Bi(111)–Ag substrate and are also visible in the following STM images.

**Figure 2:**
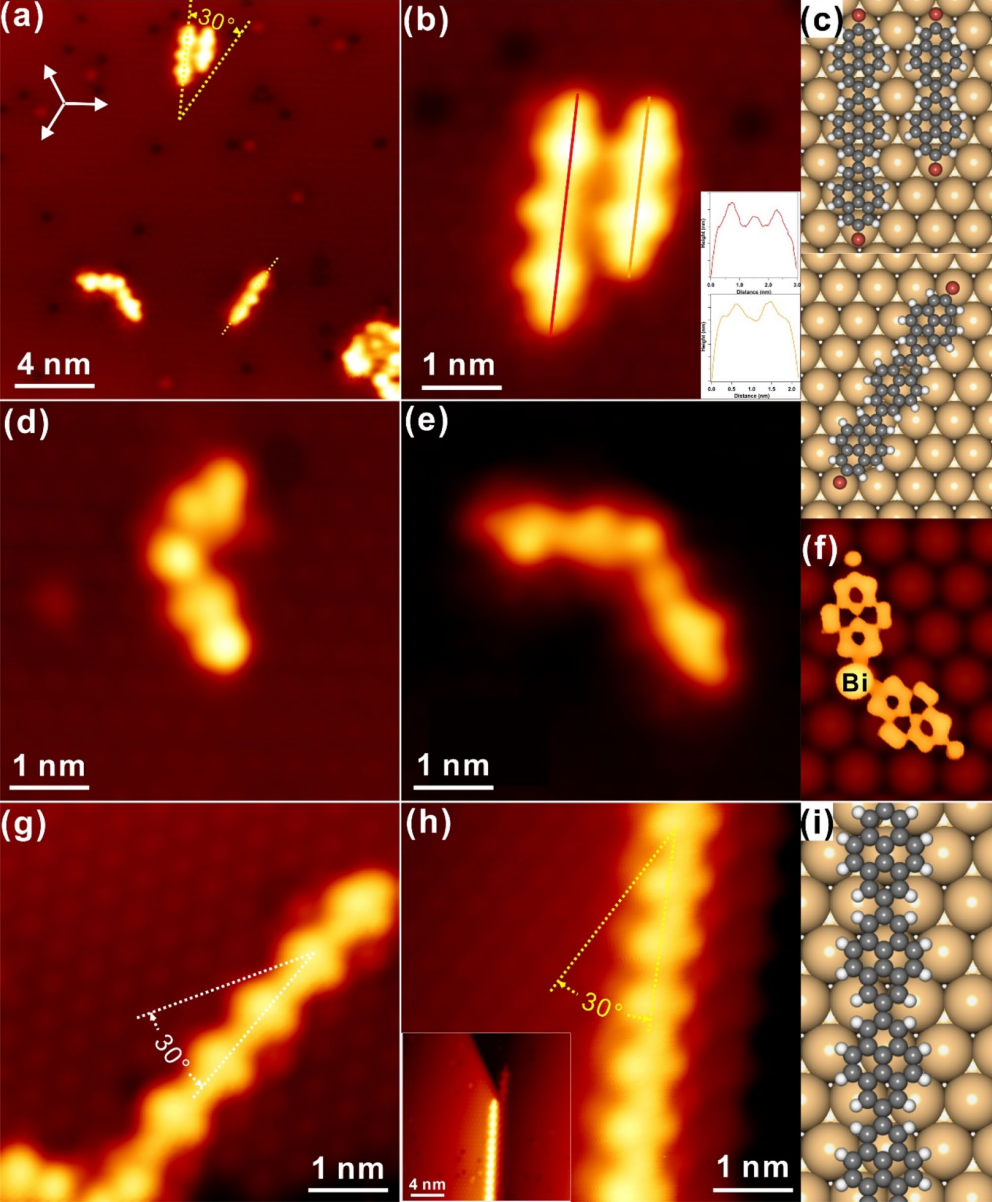
STM images recorded with step-wise annealing. **(a)** Surface topology after annealed to 380 K with the zoom-in image presented in **(b)**. **(c)** Optimized adsorption configurations for dimers and trimers observed in **(b)**. **(d,e)** Nonlinear dimer and tetramer formed after annealing to 380 K. **(f)** STM simulation (bias: − 1.5 V) of the zig-shaped dimer with Bi bound in middle from **(d)**. **(g)** The extended oligomer found after 410 K annealing. **(h)** Formation of polymer chain with annealing to 440 K and the large-scale image shown inset. **(i)** DFT optimized model for the polymer chain in **(h)**. Scanning parameters: U_bias_ = − 1.5 V, I_t_ = 0.3 nA.

Subsequent annealing to 380 K results in considerable changes of surface topography. First, disappearance of self-assembly phase is obvious with the significant desorption of precursors, as seen in Fig. 2a. Undoubtedly, the desorption of Br_2_Py shall be assigned to the weak molecule–substrate interaction mentioned before. On the other hand, interestingly, extended oligomers (for example, dimer, trimer and tetramer) with limited length are discovered after annealing regardless of the pristine inertness of bismuth atoms, and are revealed to be aligned at 30° with respect to the three-fold symmetry directions of Bi(111). In addition, the oligomer can also be found along the principal symmetry direction, as seen at the bottom of Fig. 2a highlighted with the yellow dashed line. Meanwhile, dim dots are also visible on surface which are assigned to Br atoms detached from parent molecules after the scission of the Br–C bond^19,20^. A close investigation at molecular chains is shown in Fig. 2b, and it can be resolved that dimer and trimer are formed and aligned in a manner of parallel packing with one end aligned on the Bi–Ag(111) surface. With line profiles depicted, it is discovered that the length of dimer and trimer is around 3.0 nm and 2.1 nm, respectively, which fits reasonably with the configuration of two and three covalently linked pyrene residues. Thus, no Bi atoms are expected to be bound in between pyrene residues, and the linear trimer and dimer are in fact constructed with direct C–C coupling. Based on these discussions, schematic molecular models for the dimer and trimer are depicted in Fig. 2c (with the adsorption energy of 1.4 eV as predicted by DFT calculations), whereas oligomers are arranged commensurately with substrate and the Br substitutes are placed around the hollow site, while the trimer aligned along the principal three-fold symmetric direction is also commensurately packed on the Bi(111)–Ag substrate. Besides, nonlinear oligomers (the angle between two pyrene residues is approximately 120°) are also revealed, as seen in the left bottom in Fig. 2a and other areas on surface, and are clearly presented in Fig. 2d and e, respectively. Based on previous reports for Ullmann-like coupling on coinage metal substrates, such nanostructures might be assigned to the formation of organometallic (OM) species with the semimetal adatom bound in between pyrene residues, while DFT simulation of the Bi-bound dimer delineated in Fig. 2f shows reasonable agreement with the STM observation in Fig. 2d. Nevertheless, it is also possible to form OM dimer with the Ag adatom bound in the middle which cannot be excluded unambiguously solely from STM measurements at this stage. In order to clarify this debate, comparison between Bi-bound dimer and Ag-bound dimer has also been performed, as presented in Fig S2 in ESM, whereas STM simulation of the OM dimer with Ag atom coordinated shows apparent inconsistence with the experimental observation in Fig. 2d. In addition, the adsorption energy of Ag-dimer is relatively higher that of Bi-dimer, making Ag-dimer the unfavourable configuration. Thus, one can conclude that these OM species are most probably coordinated by Bi adatoms. Thus, it is interesting to reveal the Bi-C bonding motifs in OM phases herein, which has been seldom reported before. Consequently, it can be drawn that the bismuth-silver interface indeed initiates the surface dehalogenation reaction with the inevitable desorption of Br_2_Py molecules after mild annealing, manifesting the unique characteristics of Ullmann dehalogenation on Bi(111)–Ag.

Further annealing to 410 K and 440 K, respectively, promotes sequential Ullmann-like coupling reactions and brings the formation of considerably extended oligomers and polymers, as observed in Fig. 2g,h. First, 410 K annealing greatly assists the Ullmann-like coupling and results in elongated organic oligomers without any OM species left, manifesting the relative stability of OM intermediates below 410 K annealing. More interestingly, linear chains are discovered along the step edge with significant length at annealing to 440 K as shown in Fig. 2h, which might be initially formed at other areas, diffuse over the terrace during thermal annealing, and get stabilized at the step edge. Again, the desorption of organics is apparent manifesting the weak molecule–substrate interaction. Meanwhile, it is revealed that the molecular chain is aligned 30° with respect to the high symmetry direction of Bi(111), which is similar to the situation of dimer and trimer formed after annealing to 380 K in Fig. 2a. In addition, the linear chain is uniformly organized with the peanut shape, while no metal atom can be recognized inside the chain, indicating that OM intermediates might have already been transformed into the covalently coupled nanostructures. Nevertheless, these chains are still terminated with Br atoms with the typical bright protrusion ends as generally reported in literature^43^. Notably, dissociated Br atoms from parent molecules also desorb from surface during thermal annealing, as discovered in Fig. 2g,h whereas Br atoms are only resolved in the large-scale image^23,44^. Further investigation of the adsorption/desorption behaviour of Br atoms is summarized in Fig. S3 in ESM, and it is revealed that Br atoms are robustly adsorbed on Bi(111)–Ag until 630 K annealing when desorption of all organics and Br atoms is realized and the pristine Bi–Ag surface gets recovered. Meanwhile, DFT optimized adsorption configuration for the organic chain in Fig. 2h is illustrated in Fig. 2i with the adsorption energy of 0.7 eV, which shows reasonable agreement with the STM observation in periodicity and further confirms that OM species no longer exist at this stage. Indeed, the STM topology of the organic chain is close related to the covalent linking of pyrene residues after debromination, in accordance to the typical π-conjugated aromatic systems.

**Figure 3:**
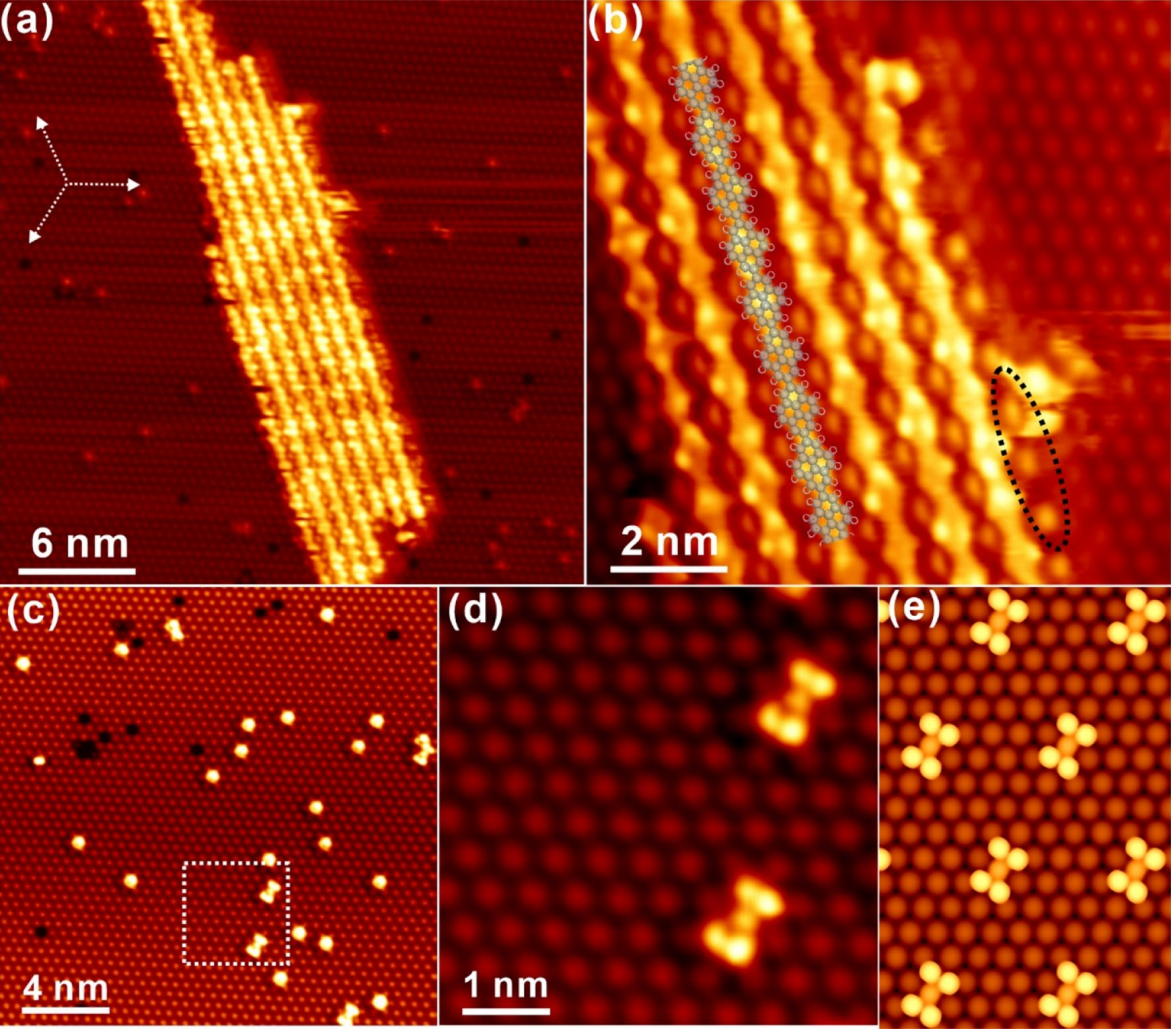
**(a)** Overview STM image taken after the deposition of Br_2_Py on the hot Bi–Ag(111) substrate kept at 530 K. **(b)** Zoom-in STM image of the polymer chains in panel **(a)**. **(c)** Overview STM image recorded on the bare Bi–Ag(111) substrate after dehalogenation and **(d)** zoom-in view of the white framed region in panel **(c)**. U_bias_ = − 1.8 V, I_t_ = 0.3 nA. **(e)** DFT simulation of Br clusters on Bi(111)–Ag at the bias of − 1.8 V.

It has been demonstrated above that the Bi(111) layer atop silver shows appealing ability in promoting the surface Ullmann-like coupling. However, due to the weak interaction between molecules and the Bi(111) surface, the amount of polymers gets less and less after the step-by-step annealing, and even vanish after annealing to a certain temperature, which makes further exploration of coupling reactions difficult. In order to elaborately investigate the catalytic activity of the thin Bi(111) layer atop Ag towards surface Ullmann-like coupling, an alternative measures has also been utilized by depositing the Br_2_Py precursor onto the preheated Bi–Ag(111) substrate. In practice, deposition of precursors on the preheated substrate has been explored at varying temperatures in this work, say, 440 K (high enough to initiate the aryl-aryl coupling as revealed in Fig. 2) and 530 K, respectively. While the preheated surface at 440 K indeed promotes the homocoupling of Br_2_Py (presented in Fig. S4 in ESM), however, the number of reacting products is not as high at that on the 530 K preheated substrate, as preheating at a higher temperature greatly enhances the dehalogenative reactivity towards coupling reactions. Thus, STM images obtained from the preheated surface at 530 K are focally discussed as illustrated in Fig. 3. It is seen from Fig. 3a that, ordered array of one-dimension (1D) chains is formed immediately upon deposition, while certain amounts of clusters are randomly distributed on bare areas of Bi(111). Apparently, these 1D chains are close packed in order by roughly following the high symmetry directions of Bi(111) at first glance. More interestingly, oligomers get incredibly extended with the length of up to 50 nm, which is much longer than those formed before via sequential annealing. Detailed inspection in Fig. 3b shows that 1D molecular chains are constructed in a well aligned manner that pyrene residues in neighbouring chains are directly facing to each other, while the pyrene frame is imaged as two non-identical bright protrusions. Besides, elongated dots are clearly resolved with the regular confinement between molecular chains, which can be assigned to liberated Br atoms from parent precursors after Ullmann-like coupling, as generally recognized in literature^18,37,45^. Meanwhile, the elongate shape of Br atoms might be related to the adsorption position (hollow site) with confinement in between linear chains on Bi(111)–Ag, causing the steric effect during STM imaging. Notably, dissociated bromine atoms are not only localized in between polymer chains on Bi(111), but they can also be stably adsorbed just on the side of linear chains, as marked by the black ellipse in Fig. 3b, which is an interesting behaviour suggesting the remarkable mediating effect on the adsorption of Br atoms from polymer chains. In addition, further investigation of the randomly distributed clusters in Fig. 3a is elaborated in Fig. 3c. While bismuth atoms from substrate are clearly resolved, it is discovered that the I-shape clusters are built on top of Bi(111), as marked by the dashed line in Fig. 3c. Detailed structure information is revealed by the zoom-in STM image in Fig. 3d. One may tentatively infer that these clusters are formed by dissociated Br atoms after C–Br bond scission, which is evidently supported by further DFT calculations illustrated in Fig. 3e where the I-shape protrusion consists of 5 Br atoms with the central one relatively dim due to the lower adsorption height. In contrast to previous reports of the accumulation of detached Br atoms into the close packed array on other metal surfaces^45–47^, herein, different observations point out the uniqueness of adsorption of Br atoms on Bi–Ag(111) and the delicate interaction between bromine and bismuth atoms. Not surprisingly, further annealing afterwards to higher temperature (for instance, 630 K) induces the desorption of polymer chains and the substrate is recovered to the pristine Bi(111)–Ag surface, as discussed before in Fig. S3 in ESI.

**Figure 4:**
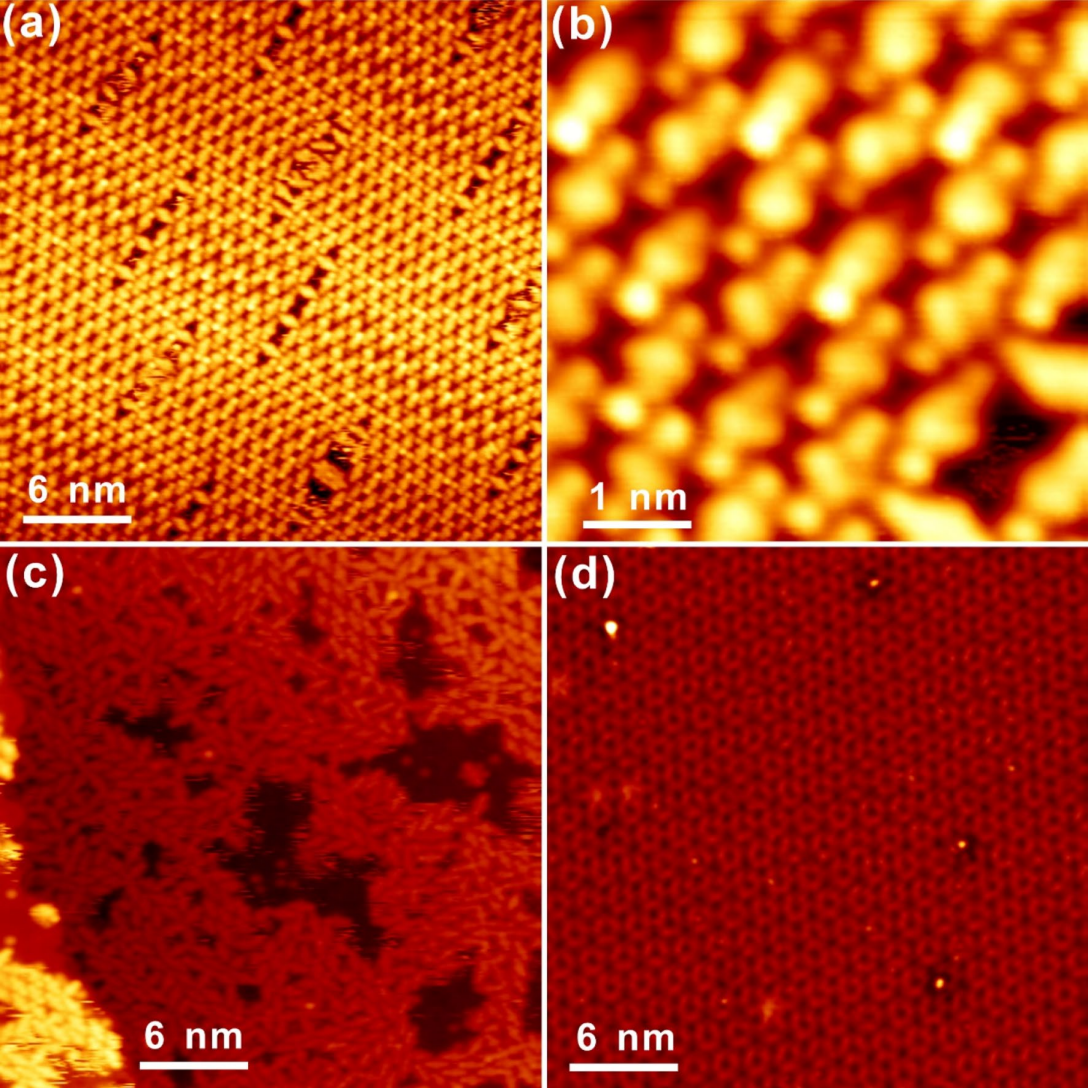
Investigation of the adsorption of Br_2_Py precursors on Bi–Au(111). **(a)** STM recording of self-assembled Br_2_Py molecules on Bi–Au(111) at RT. **(b)** Zoom-in STM view of the self-assembly from panel **(a)**. **(c)** Br_2_Py monomers are disorderly aligned on Bi–Au(111) after annealing to 350 K, while partial desorption of molecules can also be resolved. **(d)** Further annealing to 380 K inducing the complete desorption of organics, with the bare Bi–Au(111) revealed with the root 37*root 37 periodic structure. Scanning parameters: U_bias_ = − 1.8 V, I_t_ = 0.3 nA.

Dehalogenation of Br_2_Py on the Bi(111)–Ag surface has been clearly identified under different annealing conditions, manifesting the promising catalytic ability of the bismuth-silver interface towards the on-surface aryl-aryl homocoupling reaction, which in principle clarifies the ambiguous issue of bismuth’s activity supported on the silver substrate. Importantly, in order to get an unambiguous understanding of the catalytic mechanism of Bi(111)–Ag, deposition of Br_2_Py precursors on the bismuth-covered Au(111) has also been employed and is summarized in Fig. 4. Apparently, self-assembly of Br_2_Py is arranged on Bi–Au(111) as expected in Fig. 4a, analogous to that on Bi–Ag(111), with the intermolecular interaction mediated by both halogen bonding and hydrogen bonding motifs as highlighted in Fig. 4b. Interestingly, thermal annealing to 350 K induces considerable changes of surface morphology from ordered self-assembly to disorderedly parked monomers, as observed in Fig. 4c. Meanwhile, no dehalogenation coupling of Br_2_Py is discovered with the intact molecular structure well preserved, although precursors are in muddle after mild annealing at 350 K. Notably, partial desorption of organic molecules is resolved in Fig. 4c, since parts of Bi–Au(111) surface get exposed. Surprisingly, stepwise annealing to 380 K induces the complete desorption of all Br_2_Py precursors, and thus brings the pristine Bi–Au(111) surface back with the root 37 * root 37 reconstruction^34^, as exhibited in Fig. 4d. Based on this direct comparison, it can be concluded that the bismuth layer attached on Au(111) is indeed unable to initiate the Ullmann-like coupling reaction, while the promotion of dehalogenation on Bi(111)–Ag should then be correlated with the unique growth behaviour of Bi on Ag(111): by incorporating with Ag atoms on surface, bismuth atoms get alloyed with silver and thus, the Bi layer atop Ag becomes active via electron transfer from Ag^48^, inducing considerable catalytic ability towards dehalogenation coupling reactions. On the other hand, in the case of Br_2_Py atop Bi–Au(111), since the bismuth layer prefers the atomic layer growth on Au(111) without surface alloying, the pristine inert property of bismuth atom due to its tightly bound 6S^2^ electrons completely blocks the reactivity of the gold substrate underneath. Moreover, the difference in the growth mode of Bi on Ag(111) and Au(111) can be assigned to the relativistic effect (Ag(5s^1^4d^10^), Au(6s^1^4f^14^5d^10^), Bi(5d^10^6s^2^6p^3^)) and the fact that, the relative atomic radius difference between Bi and Au is bigger than 15%, which makes it unfavourable for the substitutional solid solution according to the Hume-Rothery rule^35^.

In addition, a comprehensive comparison of the dehalogenative coupling of Br_2_Py on the bare Ag(111), Bi/Ag(111) and B/Au(111) will shed more valuable insights onto the understanding of dehalogenation reaction mechanism on Bi(111)–Ag, which is summarized and illustrated in Fig. S5 in ESI. As observed in Fig. 2 and Fig. 3, reaction steps of Br_2_Py on Bi(111)–Ag involve: (1) dissymmetrical debromination of Br_2_Py; (2) formation of organometallic intermediates with the adsorption of released Br atoms from parent molecules; (3) aryl–aryl coupling towards extended organic chains with C–C bonding after further annealing; (4) desorption of bromine atoms and organic chains in the end. In contrast, dehalogenation is straightforward after adsorption of Br_2_Py on a bare Ag(111) surface at room temperature, with the coexistence of intact monomers and OM dimers. Stepwise annealing first promotes the formation of extended organometallic chains, and then results in the aryl-aryl coupling towards the ordered domain of covalent organic chains. Obviously, there are distinct differences between the Ullmann-like coupling on Bi(111)–Ag and Ag(111). First, no extensive OM chains are discovered on Bi(111)–Ag except OM dimers and OM tetramers, while long-range ordered OM chains are clearly observed on Ag(111), indicating that the pristine inert property of bismuth makes it hard to get many enough Bi-C bonds for the formation of long OM chains. Second, the desorption of reaction products (organic chains) is straightforward after stepwise annealing, and the recovery of the pristine Bi(111)–Ag surface is realized at a certain temperature annealing (630 K), while on silver or other metal surfaces, covalent organic chains usually gets destroyed before desorption and the accumulation of side produces becomes more and more visible as a function of stepwise annealing^18–21^.

Based on these observations and discussions, it has been demonstrated that Bi layer atop Ag(111) shows promising reactivity towards the Ullmann-like coupling of the halogenated molecule. However, the apparent desorption of organic nanostructures during step-wise annealing procedure points out the relatively weak interaction between the covalently coupled polymer chains and the Bi(111)–Ag substrate, as well as that between detached Br atoms and the Bi substrate. Consequently, thin bismuth layer atop Ag(111) functions as a buffer layer between organic molecules and Ag(111), and is embedded with certain activity via surface alloying, which thus will have appealing advantages to be the catalytic platform for the investigation of the Ullmann-like coupling by initiating the controllable coupling reactions and avoiding the accumulation of other side products^49,50^. With these merits, it can be inferred that bismuth layer grown on certain active metal substrates is an appealing template to elaborately investigate the mechanism of on-surface Ullmann-like coupling reactions towards rational synthesis of tailor-made organic nanostructures.

## Conclusions

In summary, we have comprehensively investigate the self-assembly and Ullman-like coupling reaction of Br_2_Py on thin bismuth layers supported on the silver substrate, and demonstrated that the Bi(111)–Ag surface could indeed initiate the dehalogenation reaction with mild annealing. By sequential heating of the self-assembled Br_2_Py molecules or depositing the Br_2_Py precursor on a hot substrate, dehalogenation and aryl-aryl homocoupling reactions are unambiguously witnessed on Bi(111)–Ag. With OM intermediates formed during reactions, covalently linked organic chains are ultimately fabricated with the considerable length. In comparison to the adsorption of Br_2_Py on Bi–Au(111), it is demonstrated that the reactivity of Bi(111)–Ag towards dehalogenation reactions is correlated with surface alloying of Bi–Ag, while atomic bismuth layer atop Au(111) cannot get alloyed due to the relativistic effect, resulting in the desorption of precursors without further dehalogenation by blocking the activity of Au(111). In addition, the weak organic-substrate interaction on Bi(111)–Ag, contributed by the pristine property of bismuth atoms, is also notable and affords the opportunity to clearly elucidate the on-surface Ullman coupling reactions. Ultimately, our report proposes an appealing platform to deeply understand reaction mechanisms of on-surface Ullman-like coupling with atomic insights towards rational synthesis of tailor-made organic nanostructures.

## Methods

STM measurements were performed in a FERMI SPM system (ScientaOmicron) with the base pressure better than 2 × 10^–10^ mbar. The sample stage in STM chamber was cooled at liquid nitrogen temperature (around 78 K) as well as the tip during scanning, while the tip was prepared by cutting Pt/Ir alloy wires. During STM scanning at the constant current mode, the bias voltage was applied to the tip and sample was grounded. STM images were processed afterward using the WSXM software (4.0 Beta, http://www.wsxm.es/download.html)^51^.

In order to get an atomically smooth Bi(111) layer on top of silver and gold substrates, respectively, a clean Ag(111)/Au(111) substrate (Mateck) was obtained first by cycles of Ar ^+^ sputtering followed with thermal annealing (650 K for Ag(111), 750 K for Au(111)), and the surface quality of Ag(111)/Au(111) was checked by STM and low energy electron diffraction (LEED). Bismuth powder (Mateck) was thoroughly degassed in an UHV evaporator (OmniVac) prior sublimation while the deposition rate was monitored by a quartz microbalance. Upon deposition on Ag(111), the Bi(110) phase was first formed at low coverage, while the Bi(111) layer was ultimately induced after a full coverage of one monolayer (ML). Evidence of the Bi(111) phase on silver can be found from the STM image in Fig. S1a, where close packed Bi atoms are clearly resolved with a hexagonal pattern, and Fast Fourier transform (FFT) in Fig. S1b of STM image. Furthermore, LEED pattern recorded on the Bi/Ag(111) surface clearly indicates the appearance of Bi(111) structure on top of Ag(111) (root 3*root 3 reconstruction)^48^ while the LEED pattern from the pristine Ag(111) is shown inset for comparison. It is thus can be concluded that Bi–Ag(111) interface has been obtained. To get the Bi- Au(111) substrate, roughly 1 ML bismuth was deposited onto Au(111), with the initial structure of 5*5 reconstruction at low coverage, and the root 37 by root 37 R25.3° periodic structures at monolayer coverage, which is usually referred as the stable phase of Bi–Au(111)^34,52^.

DFT calculations were performed using the Vienna ab initio simulation Package (VASP) under the framework of DFT^53^. The PBE functional^54^ was used in combination with the third-generation van der Waals dispersion correction due to Grimme (DFT-D3)^55^ and the projector-augmented wave (PAW) ansatz for atomic cores. A plane-wave cut-off energy of 450 eV was employed, while the k-point sampling was chosen to be 3 × 3 × 3 in order to obtain a realistic and accurate picture of the energetics. For simulation of the OM intermediate with the Bi-C bonding, a three-layer slab of the Ag(111) supercell (9 × 9) was utilized with lateral dimension of 25.96 × 25.96 Å^2^, while the vacuum layer of 10 Å was introduced to isolate the interactions of slabs from each other. The lattice parameter of the Ag(111) surface after optimization was found to be 2.88 Å, in good agreement with literature reports^56^. Afterwards, a bismuth layer was adsorbed on top of Ag(111) surface with the root 3*root 3 reconstruction, forming a Bi(111) layer with a slightly large lattice constant compared to the pristine bulk Bi(111). The bismuth layer was free together with the adsorbed OM structures during geometry optimization, while the bottom three Ag(111) layers were kept fixed. Geometries were optimized until the forces on the active atoms dropped below 0.05 eV/Å. The convergence criterion is 10^‑4^ eV in total energy difference. Simulated STM images were obtained in the framework of Tersoff-Hamann approximation with the gnuplot software (5.2, https://sourceforge.net/projects/gnuplot/files/gnuplot/5.2.8/) by plotting partial charge densities which were acquired by a static self-consistent calculation based on the DFT optimized configurations.

## Supplementary Information

Supplementary Figures.
