# Supplementary material for: Initiating Ullmann-like coupling of Br2Py by a semimetal surface
Source: Sci Rep. 2021 Feb 9;11:3414. doi: 10.1038/s41598-021-82973-z (PMC7873249; doi:10.1038/s41598-021-82973-z)
Supplement: Supplementary file 1 — Supplementary Figures. [file 41598_2021_82973_MOESM1_ESM.docx]

**Supporting Information**

**Initiating Ullmann-like coupling of Br_2_Py by a semimetal surface**

Jinping Hu^1,2^, Jinbang Hu^1,3^, Hongbing Wang^1,2^, Kongchao Shen^1,†^, Huan Zhang^1,2^, Chaoqin Huang^1,2^, Lei Xie^4^, Qiwei Tian^5^, Han Huang^5^, Zheng Jiang^1,2,4^, and Fei Song^1,2,4,*^

*^1^ Key Laboratory of Interfacial Physics and Technology, Shanghai Institute of Applied Physics, Chinese Academy of Sciences, Shanghai, 201000, China*

*^2^ University of Chinese Academy of Sciences, Beijing, 100100, China.*

*^3^ Center of Quantum Spintronics and Department of Physics, Norwegian University of Science and Technology, Trondheim, NO-7491, Norway*

*^4^ Shanghai Synchrotron Radiation Faciality, Zhangjiang Laboratory, Shanghai Advanced Research Institute, Chinese Academy of Sciences, Shanghai, 201000, China.*

*^5^ School of Physics Science and Electronics, Central South University, Changsha, 410083, China*

*^†^ Present address: Institute of Functional Nano & Soft Materials (FUNSOM), Soochow University, Suzhou, 215123, China*

***Corresponding author: Prof. Dr. Fei Song, songfei@sinap.ac.cn


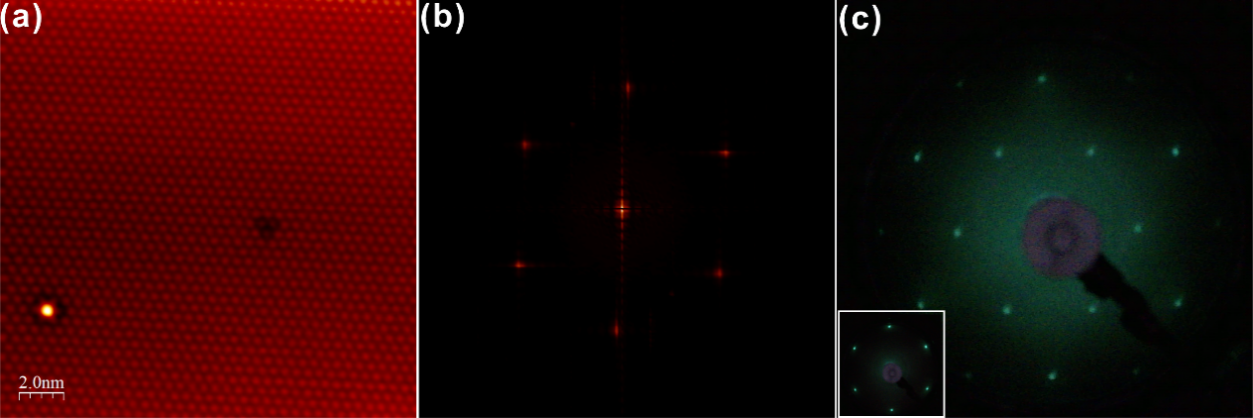


**Figure S1.** Preparation of Bi(111) on the Ag(111) substrate. (a) STM image on the Bi-Ag(111) surface (U_bias_=-1.8 V, I_t_=0.2 nA). (b) FFT of STM image in panel (a). (c) LEED on the Bi-Ag(111) surface recorded at 65 eV, while the LEED pattern from the pristine Ag(111) is also shown inset.


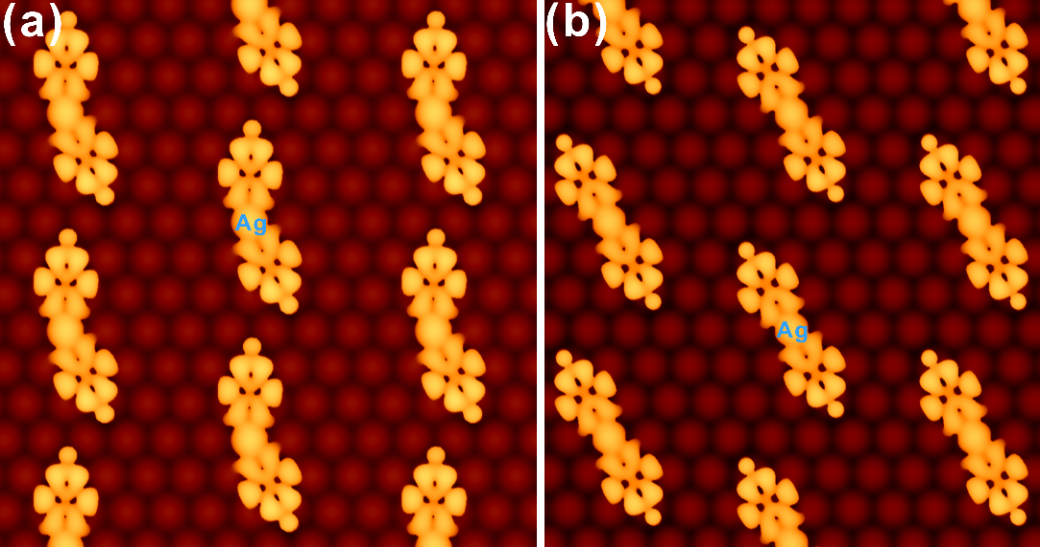


**Figure S2.** Comparison of metal coordinated dimers on Bi-Ag(111) with DFT calculations. (a) STM simulation of the nonlinear OM dimer with the Ag atom bound in the middle. (b) Ag-bound OM dimer with the linear shape predicted by DFT calculations, U_bias_=-1.5 V. Inconsistence can be found between Figure S2a and Figure 2d, whereas Ag-bound dimer is apparently expanding with a larger angle (150 °/180 °) in (a) and (b) between the two pyrene residues. Although these two configurations in (a) and (b) are both stable, the Bi-coordinated dimer proposed in Figure 2d is, however, the most favored phase from the energy point of view as well (-1176.3 eV, compared to -1175.8 eV for nonlinear Ag-bound dimer and -1175.9 eV for linear Ag-bound dimer.

**
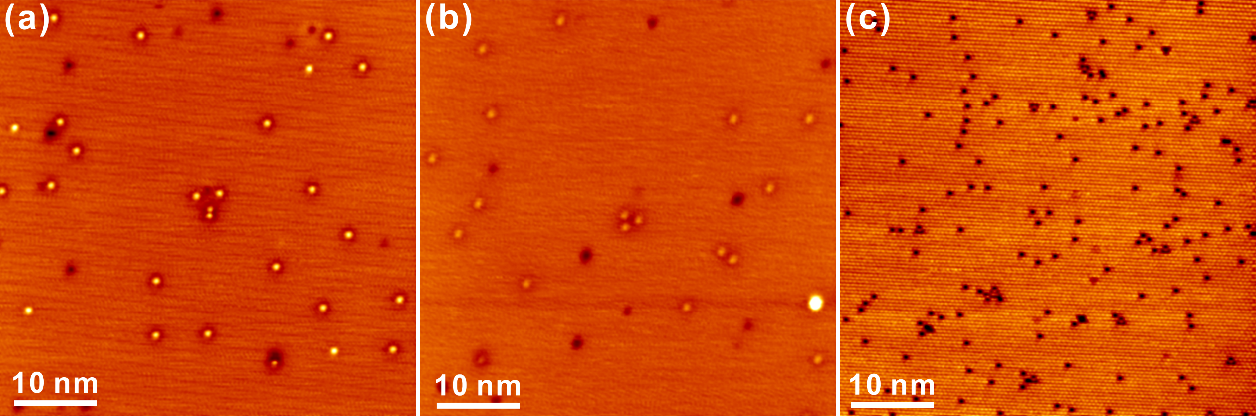
**

**Figure S3.** Evolution of the adsorption of dissociated Br atoms from parent precursors as a function of thermal annealing. (a) at 440 K (from the same sample as that in Figure 2h), (b) at 570 K annealing, the adsorption of Br atoms is still visible, and (c) after annealing to 630 K, the pristine Bi-Ag(111) surface recovered without any adsorption of Br atoms or organic nanostructures. U_bias_=-1.8 V, I_t_=0.3 nA.

**
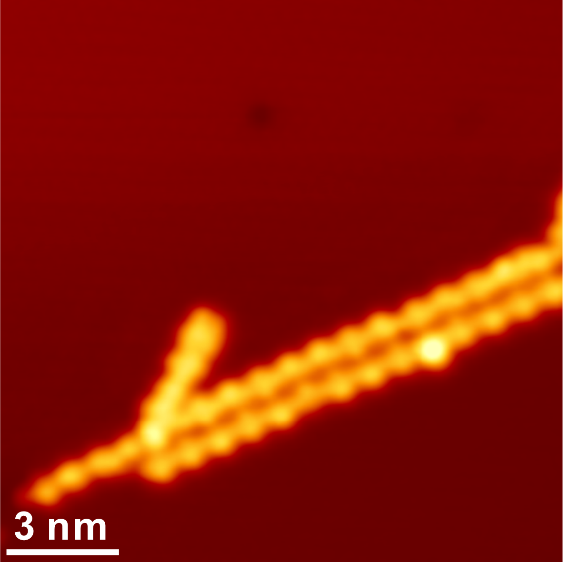
**

**Figure S4.** Formation of linear organic chains by depositing Br_2_Py precursors on the preheated Bi-Ag(111) at 440 K. The number of chains is significantly less than that on the preheated Bi-Ag(111) substrate at 530 K. U_bais_=-1.5 V, I_t_=0.2 nA.


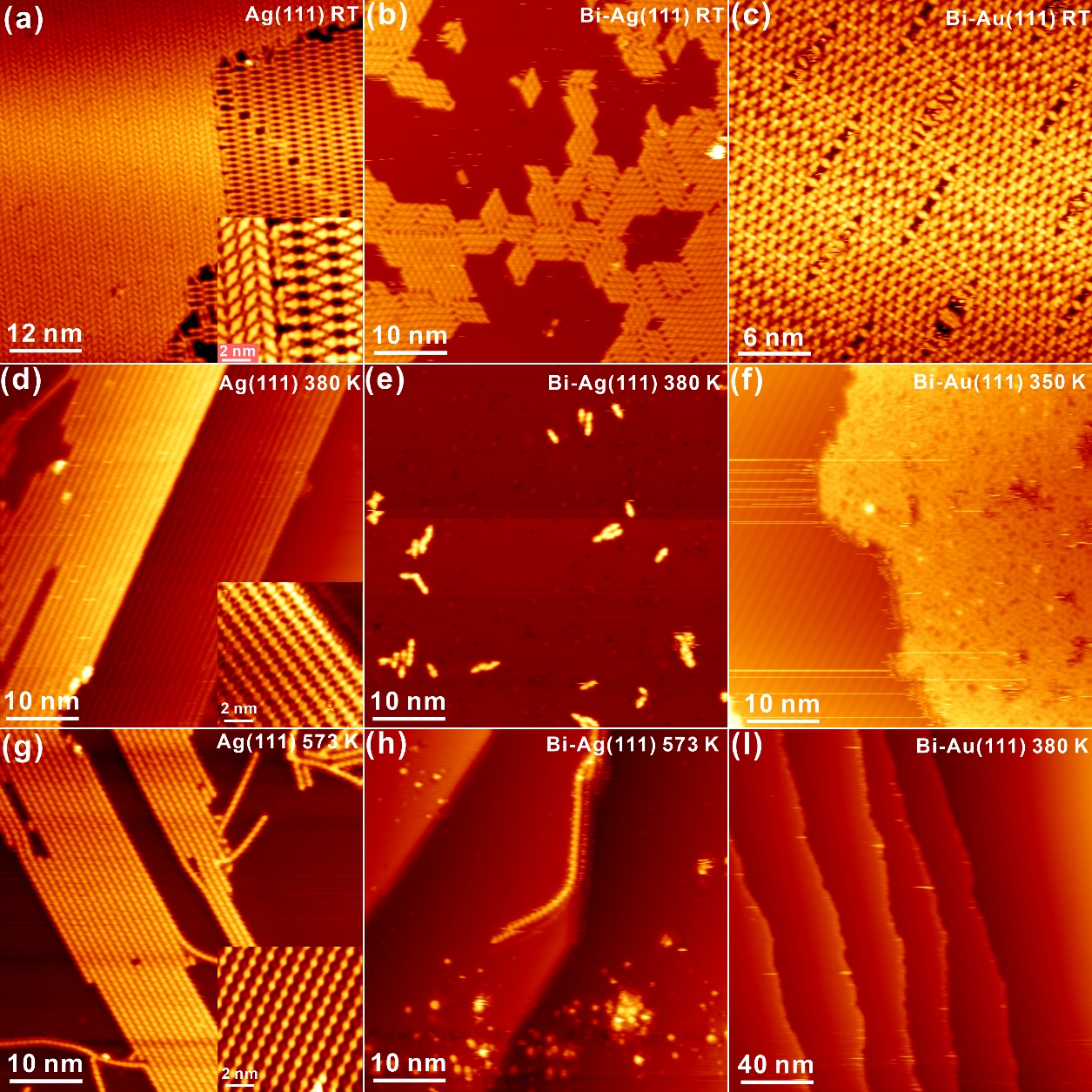
**Figure S5**. Direct comparison of Ullmann-like coupling reactions on the bare Ag(111), Bi/Ag(111), and Bi/Au(111), respectively. (a) deposition of Br_2_Py on Ag(111) at RT results in the coexistence of intact monomers and oligomers. A close-up view at the grain boundary showing the assembly of Br_2_Py molecules and organometallic dimers. (d) Thermal annealing to 380 K greatly promotes the dehalogenation of precursor and the subsequent interlinking into extensive organometallic chains. (g) Stepwise annealing to 573 K facilitates the aryl-aryl coupling and the ultimate formation of covalent organic chains with the long-range order. Large-scale STM images of dehalogenative coupling on Bi/Ag(111), (b) the self-assembly at RT, (e) annealing to 380 K, and (h) annealing to 573 K. Overview STM images of Br_2_Py precursors on Bi/Au(111) at (c) RT, (f) annealing to 350 K, and (i) annealing to 380 K with the bare Bi/Au(111) surface exposed. Scanning parameters: U_bias_=-1.5 V for the high-resolution images in the inset of (a), (d), (g) and -1.5 V for all the rest, I_t_=0.3 nA.
